# A quasi-randomized feasibility pilot study of specific treatments to improve emotion recognition and mental-state reasoning impairments in schizophrenia

**DOI:** 10.1186/s12888-016-1064-6

**Published:** 2016-10-24

**Authors:** Pamela Jane Marsh, Vince Polito, Subba Singh, Max Coltheart, Robyn Langdon, Anthony W. Harris

**Affiliations:** 1 ARC Centre of Excellence in Cognition and its Disorders (CCD), Macquarie University, Sydney, NSW 2109 Australia; 2 Discipline of Psychiatry, University of Sydney, Sydney, NSW Australia; 3 Brain Dynamics Centre, Westmead Institute for Medical Research, University of Sydney, Westmead, NSW Australia; 4 Rehabilitation Services, Cumberland Hospital, Westmead, NSW Australia

**Keywords:** Schizophrenia, Social cognition, Theory of mind, Emotion recognition, Mental state reasoning, Remediation, Social cognitive training

## Abstract

**Background:**

Impaired ability to make inferences about what another person might think or feel (i.e., social cognition impairment) is recognised as a core feature of schizophrenia and a key determinant of the poor social functioning that characterizes this illness. The development of treatments to target social cognitive impairments as a causal factor of impaired functioning in schizophrenia is of high priority. In this study, we investigated the acceptability, feasibility, and limited efficacy of 2 programs targeted at specific domains of social cognition in schizophrenia: “SoCog” Mental-State Reasoning Training (SoCog-MSRT) and “SoCog” Emotion Recognition Training (SoCog-ERT).

**Method:**

Thirty-one participants with schizophrenia or schizoaffective disorder were allocated to either SoCog-MSRT (*n* = 19) or SoCog-ERT (*n* = 12). Treatment comprised 12 twice-weekly sessions for 6 weeks. Participants underwent assessments of social cognition, neurocognition and symptoms at baseline, post-training and 3-months after completing training.

**Results:**

Attendance at training sessions was high with an average of 89.29 % attendance in the SoCog-MSRT groups and 85.42 % in the SoCog-ERT groups. Participants also reported the 2 programs as enjoyable and beneficial. Both SoCog-MSRT and SoCog-ERT groups showed increased scores on a false belief reasoning task and the Reading the Mind in the Eyes test. The SoCog-MSRT group also showed reduced personalising attributional biases in a small number of participants, while the SoCog-ERT group showed improved emotion recognition.

**Conclusions:**

The results are promising and support the feasibility and acceptability of the 2 SoCog programs as well as limited efficacy to improve social cognitive abilities in schizophrenia. There is also some evidence that skills for the recognition of basic facial expressions need specific training.

**Trial registration:**

Australian New Zealand Clinical Trials Registry ACTRN12613000978763. Retrospectively registered 3/09/2013.

## Background

Impaired social cognition is a core feature of schizophrenia [1]. Social cognition allows us to make inferences about how another person might be thinking and feeling and then to predict their likely behavior so we can successfully navigate our social world [2]. An increasing body of evidence shows that impaired social cognition is predictive of impaired social functioning in schizophrenia [3]. Social cognition is also more strongly associated with social functioning than neurocognition, and serves to mediate the relationship between neurocognition and social functioning [4]. Social functioning impairments are predictive of relapse, poor illness course, and unemployment [4, 5] and are relatively impervious to the antipsychotic drugs used to treat schizophrenia [1]. Thus, the development of psychosocial interventions to improve social cognitive functioning has emerged as a promising treatment focus in the field.

Treatments for social cognitive impairments in schizophrenia tend to be dichotomised into ‘targeted’ treatments focusing on specific impairments [6, 7] or ‘comprehensive’ treatments that treat both emotion recognition and the more complex theory of mind abilities needed to understand other people’s mental states [8–10]. Both targeted and comprehensive programs have demonstrated efficacy in improving the social cognitive domains they were developed to treat [4, 11]. Targeted emotion recognition training (ERT) uses compensatory skills-based learning (teaching where to look and how to interpret different facial movements), so the strategies used to teach emotion recognition skills are relatively more straightforward and restrained by associations between facial movements and specific expressions. In contrast, broad-based approaches attempt to improve a wider range of complex social cognitive processes, often with a focus on theory of mind (ToM) and attributional biases (i.e., mental-state reasoning abilities). Attributional biases are thought to interact with ToM impairments, particularly when situations are ambiguous, thus, exacerbating other-blaming [12]. However, the evidence to date indicates that social cognitive remediation programs show greatest efficacy for improving lower order abilities (i.e., emotion recognition) [4, 11, 13]. Moreover, emotion recognition and ToM, are differentially impaired in schizophrenia and are likely sustained by separate neural networks [14], so may require somewhat different training methods [15]. A more specifically targeted mental-state reasoning training (MSRT) may better improve higher order social cognitive abilities in schizophrenia.

Kern et al. [13] concluded that ascertaining which training methods will be most effective for improving complex higher-order abilities remains an important unanswered question. An equally important clinical question is whether ERT is a necessary building block to improve mental-state reasoning abilities; that is, can MSRT be effective when used without any ERT? Only by investigating the effects of each type of training separately can we begin to answer these important theoretical and clinical questions. Such questions have cost-benefit implications as well; targeted group training will be briefer and delivered to several people at 1 time so less clinician time is required for each participant and it is likely to be easier to maintain participant motivation to enter into, and then to remain in treatment.

Towards this end, we have already piloted a targeted 1-h ERT program with promising results [7, 16]. In those studies, we used Ekman’s Micro Expression Training Tool CD (METT; http://www.paulekman.com/micro-expressions/) and found improved emotion recognition in people with schizophrenia that was durable 1 month after testing. The METT uses a series of videos showing facial expressions with verbal commentary to direct attention to relevant facial features of commonly confused emotional expressions (e.g., using the eyebrows to distinguish fear from surprise). Following from this we piloted a novel 6-week “SoCog-MSRT” program that targets ToM and attribution style, with no specific reference to emotion recognition [8]. In this open clinical trial of SoCog-MSRT we found pre- to post-treatment improvements on a classic false-belief test of ToM [17], a test that requires the decoding of complex emotions from viewing only the eye-regions of a face (the Reading the Mind in the Eyes test; RMET) [18], and a self-report measure of social understanding [19]. However, recognition of basic facial emotions (happy, sad, angry, surprised, fearful, disgusted, neutral), shown in 100 % and 75 % morphed intensity expressions and presented as still photographs of whole faces, did not improve from pre- to post-treatment. This is most likely because in SoCog-MSRT we focus on inferring complex mental-states rather than specifically targeting the decoding of basic emotional expressions. In contrast, Bora and colleagues [20] defined the RMET as a test of mental-state decoding that does not rely solely on decoding of basic facial expressions of emotion but also encompasses ToM skills. In their study, they found that the RMET was a better predictor of social functioning outcomes than mental-state reasoning. Thus, improving mental-state decoding abilities might have important implications for real-world social functioning.

The objective of this current study was to investigate the acceptability, feasibility and limited efficacy of 2 programs to improve social cognition in schizophrenia: the above-mentioned SoCog-Mental-State Reasoning Training (SoCog-MSRT) and a newly developed SoCog- Emotion Recognition Training (SoCog-ERT) program that builds upon and extends the theoretical underpinnings of our previous work using the METT. We predicted that participants would accept the programs and find them enjoyable and beneficial? and they would be motivated to attend training sessions. We predicted that limited efficacy would be evidenced by improved basic emotion recognition following SoCog-ERT and improved mental-state decoding and mental-state reasoning following SoCog-MSRT.

## Method

### Trial design

The study was designed as a randomised controlled trial adhering to CONSORT guidelines [21] with both an active control treatment (social activities) and a wait-list control. Unfortunately, this design failed due to a number of issues pertaining to the nature of the active control, which was rejected by the study participants because they realised they were not receiving active treatment. After the cessation of the active control treatment, we were unable to recruit sufficient participants into the wait-list control group within the period available to conduct the study. This was partly due to the refusal of 1 referral site to randomise subjects in the study because the service has a strong focus on rehabilitation and wanted all patients to receive treatment.

### Randomisation

**Fig. 1:**
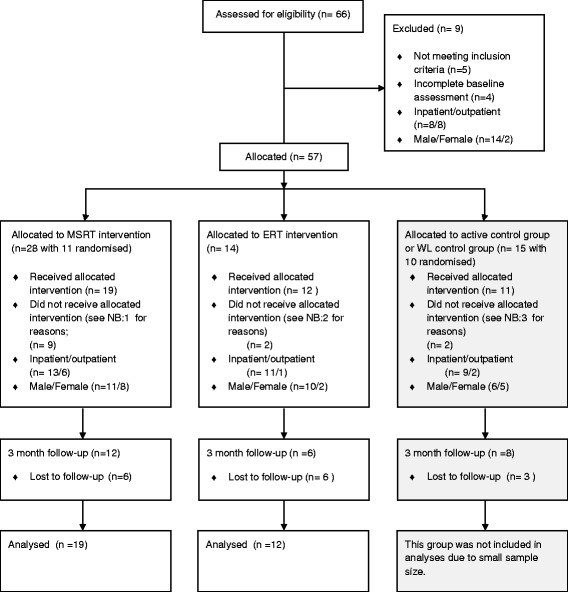
CONSORT Diagram

There was a slow rate of appropriate referrals into the study with an initial referral rate of 5–6 participants per training cohort which then reduced to as low as 2 per cohort. As such, we were limited to randomising only the first 4 cohorts of the study into either MSRT or a control group (see Fig. 1) so that we could continue to produce workable group sizes. As a result, this report will focus on 31 participants who completed 6 weeks of SoCog-ERT or SoCog-MSRT followed by post-treatment assessment. Eighteen participants were followed up at 3-months. Participants who were randomised were allocated to either MSRT or the control group in blocks of 4 with a 1:1 allocation by drawing a ticket from an opaque envelope. Nursing staff who were independent of the research study assessors administered the allocation procedure. Assessors were not blind to group as they also conducted training. Every effort was made to blind participants to the type of training (ERT or MSRT) they were undertaking.

### Participants

Inclusion criteria were a diagnosis of schizophrenia or schizoaffective disorder (chronic or acute phase), good English skills, aged 18 – 55 years and the capacity to give informed consent. Diagnosis was confirmed via medical records and/or referring clinicians. Exclusion criteria were learning difficulties, bipolar disorder or comorbid neurological illness, history of head injury (unconscious > 1 h), current substance/alcohol abuse, the presence of acute delusions that were significant enough to interfere with participation, or electroconvulsive therapy within the past 6 months.

Participants were recruited from across inpatient rehabilitation services, a forensic psychiatry ward, an outpatient Community Housing Implementation Programme, and from a community service for young people with a psychotic illness. Written referrals of patients who met the study criteria were sought from treating clinicians (psychiatrists, psychologists and nursing staff) using a checklist of inclusion/exclusion criteria. Referred patients were then approached by research staff to invite them into the study. Participation was entirely voluntary and only those patients referred by treating staff were invited to participant. Participants in the active control group and the waitlist control were offered treatment after they completed follow-up testing at 3 months.

### Ethics, consent and permissions

Following a full description of the study, either read to them by a researcher or read by them, all participants gave written informed consent. The study was approved by the Western Sydney Local Health District Human Research Ethics Committee (no. HREC09/WMEAD/36). We reimbursed participants $15AUD for each assessment (baseline, post-test and 3-month follow-up). We did not give reimbursement for the 6 weeks of SoCog training.

### Outcome measures

#### Symptoms

To control for potential effect of symptoms on treatment outcomes, current symptoms were assessed using the Scale for the Assessment of Positive Symptoms (SAPS) [22] and Scale for the Assessment of Negative Symptoms (SANS) [23] at T1 (baseline). PJM or trained research assistants conducted interviews. Research assistants were all trained using the SAPS/SANS manual and the same training videos under the supervision of experienced raters.

#### Neurocognition

To minimise testing and the potential for fatigue effects from a long test battery, we chose just 3 neurocognitive domains from the MATRICS Consensus Cognitive Battery [24] to control for changes in general cognitive performance. We chose these domains because in our previous work we have found associations between working memory and improved social cognition following ERT [7, 8]. Problem solving abilities are also essential skills for adapting to change in the environment and might affect the ability to benefit from treatment; likewise, speed of processing, important for staying on task, is associated with a range of social outcomes [25]. Premorbid IQ is predictive of long-term social outcomes [26] and was included for this reason. The neurocognitive battery comprised: 1) The National Adult Reading Test (NART) [27] to assess premorbid IQ; 2) the Digits forwards and backwards subtests from the Wechsler Adult Intelligence Scale [28] to assess working memory; 3) Delis–Kaplan Executive Function System Sorting Test (to reduce the number of variables in analyses only confirmed correct sorts scaled scores are reported) [29] to assess problem-solving abilities; and 4) the Symbol Digits Modalities Test [30] to assess speed of processing.

#### Feasibility and acceptability

Feasibility was quantified using group attendance and attrition rates. The Intrinsic Motivation Inventory for Schizophrenia Research (IMI-SR) [31] was used to assess acceptability in terms of participants’ subjective interest and enjoyment of the activity. The IMI-SR is a self-report assessment with 21 items that take around 5 min to complete and includes 3 subscales to assess participants’ subjective interest and enjoyment of the activity (e.g., I think this activity is quite enjoyable), sense of choice about doing the activity (e.g., “I had some choice about doing this activity”) and perceived value or usefulness of the activity (e.g., “I would be willing to do this again because it has some value to me”). Importantly this instrument is suitable for individuals with at least a fourth-grade reading level [31]. It is scored on a 7-point Likert scale from 1 (“not at all true) to 7 (“very true”). As the IMI-SR was specifically adapted for use in schizophrenia it was chosen as the most suitable measure of participant interest, enjoyment and satisfaction from their engagement with the treatment programs. The IMI-SR has demonstrated test-retest reliability (.77 total scores, .74 interest/enjoyment subscale, .76 choice subscale, and .70 value/usefulness subscale) following a 4-week interval. Internal consistency is also good (alpha = .92). To produce scores for enjoyment, choice, and value, we summed items across each subscale producing a range from 0 to 49; higher scores represent higher levels of enjoyment, choice, and perceived value. Total scores range from 0 to 147.

#### Social cognition

We used 4 measures of social cognitive abilities. Basic facial emotion recognition was assessed using video clips of real-life vignettes of social interactions from the Emotion Evaluation Task component of The Awareness of Social Inference Test (TASIT) [32]. This test is used to assess recognition of emotions presented in short, videotaped vignettes of professional actors communicating emotions in everyday situations comprising 28 scenes across 2 versions (A and B); we divided A and B into 2 tests of 14 items, each comprising 2 exemplars of happiness, sadness, disgust, surprise, anger, fear, and neutral. This allowed us to counterbalance this test across Time using Latin Squares. Test-retest reliability ranges from 0.74 – 0.88 and alternate forms reliability ranges from 0.62 – 0.83 [32].

The Reading the Mind in the Eyes Test (RMET) was used to assess social-perceptual aspects of ToM involving the attribution of complex mental states (e.g., compassion) [18, 33]. In our first pilot study of SoCog [8] we used the adult version of the RMET [18] but participants reported difficulty understanding the words used to describe the emotions in that version; thus, in this current study we changed to the child version which was developed to use the same emotions but with an easier-to-understand vocabulary [34]. The RMET comprises 28 photographs of the eye region of the face and the participants’ task is to pick which of 4 words best describes what the person in the photo is thinking or feeling. Each item is scored as ‘correct’ or ‘incorrect’ with correct scores summed to produce a score out of 28. Although specific information about practice effects is not available for the child version, there are no reported learning effects for the adult version when the test is repeated over time [35].

The Picture Sequencing Task [17] is a classic measure of non-verbal ToM. Specifically, this task assesses the ability to accurately sort cartoon images to tell a story about a cartoon character who has acted on a false-belief. From the Picture Sequencing Task, we used the False Belief (PSTFB) stories to assess non-verbal ToM and the Mechanical Control (PSTC) stories to test (non-social) physical cause and effect reasoning. Stories are presented in 4-card picture sequences using a simple black-and-white cartoon style. The cards are placed face down in front of participants who are asked to turn the cards over and to place them in the correct order to show a logical sequence of events. Scores range from 0 to 6. Support for the test-retest reliability of the Picture Sequencing Task is provided by an earlier treatment study that tested the efficacy of cognitive behavioural therapy in delusional individuals and found no differences from pre- to post-treatment for the false belief or control stories [36].

#### Attributional style

Attributional Style was assessed using the Internal, Personal and Situational Attributions Questionnaire (IPSAQ) [37]. We divided this into 2 subtests of 16 items each to allow counterbalancing across time. Two cognitive bias scores were calculated: Externalising Bias (number of internal attributions for positive events - number of internal attributions for negative events) score and Personalising Bias (number of personal attributions for negative events ÷ sum of personal and situational attributions for negative events) scores [37]. A Personalising Bias > .5 indicates external attributions that are more biased toward personal rather than situational explanations; a positive externalising bias indicates a tendency to blame others for negative events. Test-retest reliability is not available for this measure but it has good internal consistency (range: 0.61–76 for each subscale) [37].

### Overall treatment approach

Both SoCog programs (ERT and MSRT) consisted of 12 1-h sessions over 6 weeks. Two facilitators ran training in small groups of 3−6 participants using a manual-driven suite of novel activities and games [38]. The training approach of SoCog provides repeated exposure and practice of the skills that underlie complex mental-state reasoning abilities or emotion recognition abilities as described below.

A weekly points system with prizes is used to provide extrinsic motivation [39]. When a participant wins a game, or they contribute a valid hypothesis or observation, the facilitator can award points that are tallied at the end of each week. The participant with the most points at the end of each week then wins a prize (e.g., small toiletries and stationery items). Likewise, intrinsic motivation plays an important role in overcoming the core motivational impairments in schizophrenia [40]. With the latter in mind, we structured SoCog sessions to give a sense of control over training and to enhance engagement with the treatment. Thus, facilitators set the activity for the first 20 min of a session and then participants choose an activity for the second 20 min with a 10-min break between the first and second activity.

### SoCog-mental state reasoning training (SoCog-MSRT)

The specific training approach of SoCog-MSRT is that participants receive repeated exposure and practice of the skills that underlie complex mental-state reasoning abilities [38]. In SoCog-MSRT, this interactive model uses the spontaneous discussion of participants’ own experiences as a platform upon which to extend treatment and improve generalisation. Moreover, it allows facilitators to explore a range of possible hypotheses for beliefs, perceptions, and behaviours that will involve the group in developing a reasonable explanation for a particular situation. This further allows for different possible interpretations and inferences from all group members and presents an invaluable learning experience about often-ambiguous real world social interactions. Thus, SoCog-MSRT allows for the discussion of potentially conflicting beliefs and uncertainty about social cues. The idea is that the program focuses on teaching participants that we can make inferences about another’s thoughts, feelings, and behaviour but that these are only hypotheses that may turn out to be right or wrong. In this way, we aim to move participants away from jumping to conclusions and into the more reflective processes required for adequate real world social reasoning. Thus, activities centre on vignettes of social situations with a focus on making inferences and predictions about characters’ thoughts, feelings, and behaviours. We repeat similar vignettes across different activities with frequent repetition of training materials and concepts. Facilitators’ guide discussion and explore a range of possible hypotheses for beliefs, perceptions, or behaviours to involve the group in developing a reasonable explanation for a particular situation.

### SoCog-emotion recognition training (SoCog-ERT)

In this treatment, we combined the METT with other novel activities to reinforce information about recognising emotions. We used the METT in a collaborative group setting, rather than individually, with facilitators guiding group participants through each stage of the training CD and repeating the training videos at least 3 times during the course of treatment. We designed other new activities/games to reinforce and build on the information about salient facial features. Specifically, novel materials consist of a range of card games, board games and computer games that direct participants’ attention to the importance of facial features (eyes, nose, and mouth) and how they move to distinguish between commonly confused facial expressions [38]. Thus, similar to SoCog-MSRT, SoCog-ERT comprised repeated exposure and practice of the skills that underlie emotion recognition.

### Data analyses

To investigate limited efficacy, participants were assessed at 3 time points; baseline (T1), post-test (T2), and 3-month follow-up (T3). To minimise the number of contrasts in this acceptability and feasibility study, we examined difference scores for the MSRT and ERT groups on the dependent variables comprising the TASIT negative emotions, RMET, PSFBT, PSCT, and IPSAQ PB using the Exploratory Software for Confidence Intervals (ESCI) [41] for the following contrasts:

(A) T2 versus T1, to ascertain whether there were immediate post-training improvements on social cognitive variables;
(B) T3 versus T2, to ascertain (a) durability of any improvements found above (i.e., T3 = T2); and (b) improvements at 3-month follow-up (i.e., T3 > T2). Previous emotion recognition training studies have found improved emotion recognition of more complex stimuli were evident only one month after training and not immediate post-training [7]; thus, whereT1 did not differ from T2 (i.e., T2 = T1 indicating no immediate improvement), we tested for similarly delayed improvements at 3-month follow-up (i.e., T3 > T1).

Consistent with the guidelines published in the sixth edition of the *American Psychological Association (APA)* Publication Manual [42] we interpreted the mean of the differences on each dependent variable and the Confidence Intervals (CIs) on these mean differences and effect sizes. Specifically, we focused on whether the CI of the mean difference captured zero as a test of no effect of treatment [43]. Results are presented graphically as recommended by the APA Task Force [44]. Figures showing results were produced using ESCI Data Paired.

We used ESCI [41] to calculate unbiased estimates of effect size using Cohen’s D (D_unb_). This software uses Hedge’s formula where the adjustment factor depends on *df* and provides an unbiased estimate of effect size for small samples [41, 45]:

$$ {\mathrm{d}}_{\mathrm{unb}}=\left(1-3\ /\ {4}_{\mathrm{d}\mathrm{f}}-1\right)\ \mathrm{x}\ \mathrm{d}\ \mathrm{where}\ \mathrm{d}\ \mathrm{is}\ \mathrm{calculated}\ \mathrm{a}\mathrm{s}\ \mathrm{d}={\mathrm{M}}_{\mathrm{d}\mathrm{iff}}/{\mathrm{S}}_{\mathrm{av}} $$

## Results

### Recruitment and participant flow

Recruitment for the study began in July 2009 and ended in October 2012. Final follow-up concluded in late 2012. The recruitment process into the study is shown in the Consort Diagram in Fig. 1.

Dropouts were for the following reasons:

1. 1 participant showed paranoid delusions and 1 delusions of reference during training which were severe enough to infer with training and group/computer activities, 1 participant was annoyed that other group members were always late, 5 gave no reason for withdrawal.
2. 1 participant was discharged and the other started an English course at TAFE
3. 1 participant was discharged, 1 gave no reason

### Baseline data

Clinical, demographic and neurocognitive results are shown in Table 1. There were no gender differences on any demographic, neurocognitive, or social cognitive baseline measure. The mean age of participants was 35.55 (SD = 10.01), with a mean of 12.49 years of education (SD = 3.31). Overall measures of positive and negative symptom severity were calculated by taking the mean of the global scores from the Scales for the Assessment of Positive [22] and Negative [23] Symptoms of Schizophrenia (SAPS and SANS: range 0−5). Mean positive symptom severity was 1.39 (SD = 1.21) and mean negative symptom severity was 3.02 (SD = .93). Participants in the 2 treatments did not report any differences on the IMI-SR for their level of enjoyment, sense of choice, or perceived value of the treatments.

**Table 1:** Baseline clinical, demographic and neurocognitive scores for SoCog-MSRT and SoCog-ERT groups

Mean (SD)	SoCog-MSRT	SoCog-ERT
Age	35.94 (10.57)	34.08 (9.47)
Years of education	12.06 (2.25)	13.38 (4.72)
Chlorpromazine equivalent	519.7 (203.76)	590.45 (163.42)
NART full IQ equivalent	104.18 (7.88)	102.67 (5.68)
WAIS Digit Span Converted	8.00 (2.14)	7.33 (1.56)
Symbol Digit	-1.92 (.86)	-2.07 (.917)
DKEFS Sort	6.72 (3.01)	6.92 (2.68)
SAPS	1.32 (1.19)	1.44 (1.56)
SANS	2.87 (.98)	3.11 (1.09)

Compared to inpatients (M = -2.13, SD = .883), outpatients (M = -1.48, SD = .76) showed a trend for faster baseline speed of processing on the Symbol Digits Modalities Test [30] (t(40) = -1.909, *p* = .058). There were no other differences between inpatients and outpatients

### Acceptability and feasibility

Participants in the MSRT group attended 89.29 % of sessions and the ERT group 85.42 % of sessions. Dropout from T1 to T2 was highest for SoCog-MSRT at 32.14 % compared to 14.29 % for SoCog-ERT. The higher dropout rate for the MSRT group is consistent with lower total scores on the IMI-SR for this group (109.43 out of a possible 147; SD = 18.79) compared to the ERT group (115.2; SD = 21.23) suggesting qualitatively lower levels of acceptability for SoCog-MSRT. A breakdown of scores across the IMI-SR subscales of enjoyment, perceived choice and perceived value are shown in Table 2.

**Table 2:** Mean scores (SD) for Subscales of the Intrinsic Motivation Inventory Revised for Schizophrenia Research [31] rated at Time 2

IMI-SR subscale	Group	
	SoCog-MSRT	SoCog-ERT
Choice	41.14 (5.97)	40.70 (6.06)
Enjoyment	35.71 (7.67)	37.90 (6.84)
Perceived Value	32.57 (9.23)	36.60 (10.89)

### Social cognition

**Fig. 2:**
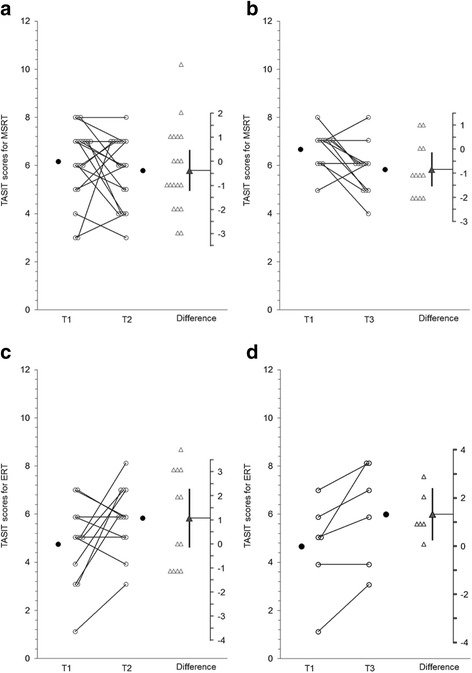
**a**, **b** MSRT group results for The Awareness of Social Inference Test (TASIT) negative emotions. **c** and **d**: ERT group results for The Awareness of Social Inference Test (TASIT) negative emotions

**Fig. 3:**
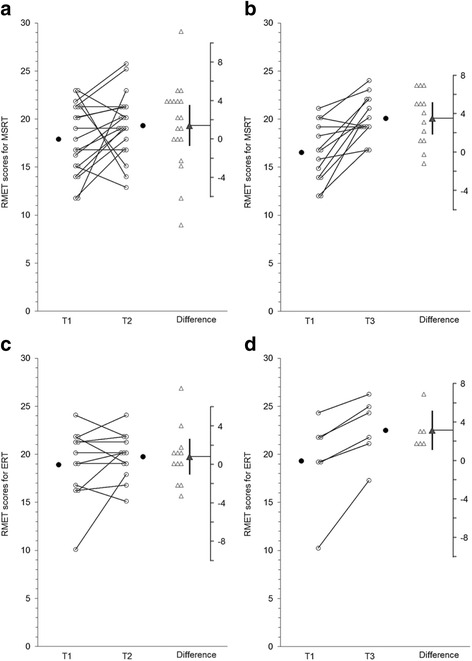
**a**, **b** MSRT group results for The Reading the Mind in the Eyes Test (RMET). **c** and **d**: ERT group results for The Reading the Mind in the Eyes Test (RMET)

**Fig. 4:**
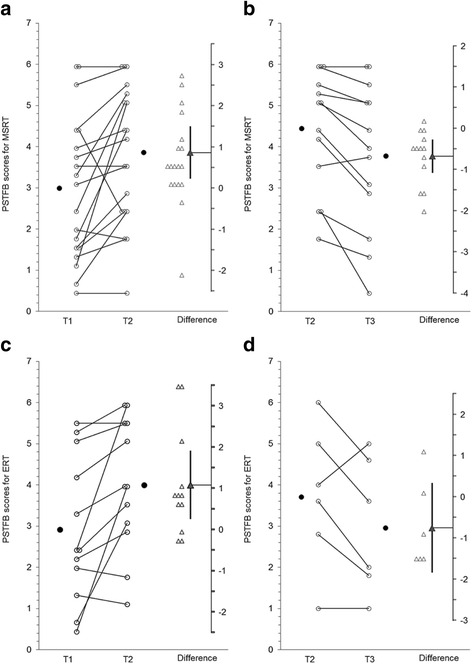
**a**, **b**: MSRT group results for The Picture Sequencing Task False Belief (PSTFB). **c** and **d**: ERT group results for The Picture Sequencing Task False Belief (PSTFB)

**Fig. 5:**
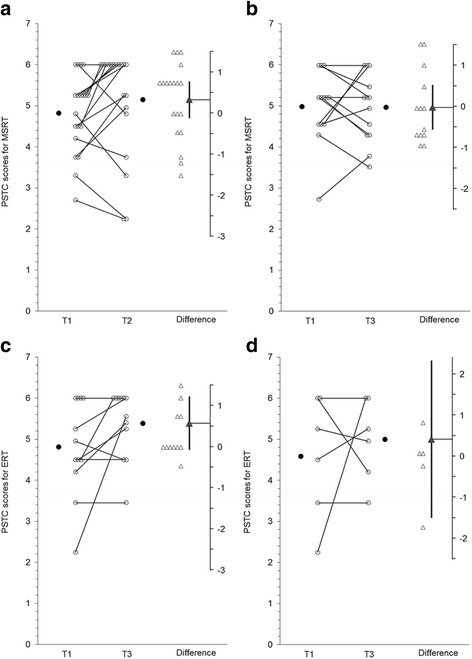
**a**, **b**: MSRT group results for Picture Sequencing Task Control (PSTC). **c** and **d**: ERT group results for Picture Sequencing Task Control (PSTC)

**Fig. 6:**
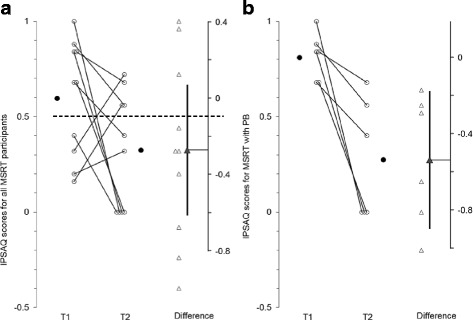
**a**, **b**: MSRT group results for the Internal, Personal and Situational Attributions Questionnaire (IPSAQ Personalising Bias: PB)

Graphs produced using ESCI Data Paired showing group data on each outcome measure are presented below. For Figs. 2, 3, 4, 5, and 6 graphs are presented as follows:

- Graph (a) shows results for the MSRT group from T1 to T2;
- graph (b) shows the MSRT group results from T2 to T3 (i.e., whether there was any immediate improvement after treatment) or where relevant T1 to T3 (i.e., whether there was a delayed improvement);
- graph (c) shows the ERT group results from T1 to T2; and
- graph (d) shows the ERT group results from T2 to T3 (durability of any improvements from T1 to T2) or T1 to T3 (delayed improvements in the absence of immediate improvements from T1 to T2).

Results are presented below for each dependent variable. All CIs presented in the results represent the 95 % CIs for the paired difference scores on the contrast of interest (e.g., T2 – T1).

#### The Awareness of Social Inference Test (TASIT) to assess emotion recognition

Figures 2a, b, c and d show group results for the Emotion Evaluation Task component of the TASIT. There was no significant change on the recognition of negative facial expressions using the TASIT (t(18) = -.91, *p* = .38, 95 % CI [-.1.22, .48] d_unb_ = -.24, see Fig. 2a) and at 3 months there was a decrease in accuracy (t(11) = -2.59, *p* = .025, CI [-1.54, -.13], d_unb_ = -.85, see Fig. 2b) after the removal of 1 outlier (defined as difference score = +5). The ERT group showed a trend for a moderate increase in recognition accuracy from T1 to T2 (t(11) = 1.95, *p* = .078, CI [-.14, 2.31], d_unb_ = .68, see Fig. 2c) with half of the sample showing increased scores, 2 no change and 4 a decrease of -1. However, the CI on the difference scores did capture zero on the difference axis therefore, it was appropriate to investigate whether there was a delayed improvement. As shown in Fig. 2d, there was an increase from T1 to T3 (t(5) = 3.16, *p* = .025, CI [.25, 2.42], d_unb_ = .54).

#### The Reading the Mind in the Eyes Test (RMET) to assess mental-state decoding

Figure 3 a, b, c and d show group results for the RMET. There was no immediate change in RMET scores for the MSRT group (t(18) = 1.42 *p* = .18, 95 % CI [-.07, 3.55], d_unb_ = .394, see Fig. 3a) but there was a delayed increase from T1 to 3-months (t(12) = 4.63, *p* = .001, d_unb_ =1.26, CI [1.87, 5.20], see Fig. 3b). Likewise, the ERT group showed no change from T1 to T2 (t(11) = .98, *p* = .349, CI [-1.04, 2.71], d_unb_ = .246, see Fig. 3c) with delayed increases from T1 to T3 (t(5) = 4.0, *p* = .01, CI [1.13, 5.50], d_unb_ = .63, see Fig. 3d).

#### Picture Sequencing Task False Belief (PSTFB) to assess non-verbal ToM

Figures 4a, b, c and d present group results for the PSTFB. Although there were increases in false belief reasoning scores for the MSRT group from T1 to T2 (t(18) = 2.87, *p* = .01, d_unb_ = .49, 95 % CI [.23, 1.51], see Fig. 4a) this regressed back toward the baseline mean from T2 to T3 (t(13) = -3.65, *p* = .003, CI [-1.07, -.27], d_unb_ = -.38, see Fig. 4b). Unexpectedly we also found increases on the PSTFB task for the ERT group from T1 to T2 (t(11) = 2.87, *p* = .015, CI [.25, 1.91], d_unb_ = .71, see Fig. 4c) and these changes were maintained from T2 to T3 (t(5) = -1.77, *p* = .137, CI [-1.84, -.75], d_unb_ = -.37, see Fig. 4d).

#### Picture Sequencing Task Control (PSTC) to assess physical cause and effect reasoning

Figures 5a, b, c and d present group results for the PSCT. There was no change in cause and effect reasoning for the MSRT group from T1 to T2 (t(18) = 1.55, *p* = .14, 95 % CI [-.12, .78], d_unb_ = .28, see Fig. 5a) or from T1 to T3 (t(12) = -0.08, *p* = .94, CI [-.56, .52], d_unb_ = -.02, see Fig. 5b). Likewise, for the ERT group there was no change from T1 to T2 (t(11) = 1.94, *p* = .078, d_unb_ = .53, see Fig. 5c) or from T1 to T3 (t(5) = .56, *p* = .60, CI [-1.50, 2.34], d_unb_ = .28, see Fig. 5d). These results suggest that the initial increased PSTFB scores was not likely to have been simply a practice effect although this cannot be ruled out given the lack of a control group.

#### The Internal, Personal and Situational Attributions Questionnaire (IPSAQ) to assess attributional style

Figures 6a and b presents group results for the IPSAQ. Data was only available for the MSRT group from T1 to T2 on this measure, with no data available for the ERT group^1^. When we examined the results for all MSRT participants we did not find any reduction in personalising biases from T1 to T2 (t(10) = -1.788, *p* = .11, d_unb_ = -.83, see results in Fig. 6a). However, only a subset of individuals with schizophrenia showed a PB, defined as a score > .5 [34], and including individuals without an existing PB at baseline might serve to mask potential improvements [46, 47]. Thus, we then focussed our analyses solely on those participants with a baseline PB score > .5. Results showed a significant decrease from T1 to T2 (t(6) = -3.82, *p* = .01, CI [-.90, -.18], d_unb_ = -1.9, see Fig. 6b).

## Discussion

The aim of this study was to explore the feasibility, acceptance, and limited efficacy of 2 novel social-cognitive remediation (SoCog) programs to improve social cognition in schizophrenia. We predicted that participants would accept the program and find it enjoyable and beneficial and they would be motivated to attend training sessions. We further predicted that following training, the SoCog-ERT group would show enhanced recognition of basic facial expressions of emotion whereas the SoCog-MSRT group would show improved mental-state decoding and mental-state reasoning.

### Feasibility and acceptability

Attendance rates were better than in similar studies (e.g., 65 % [48] and 79.17 % [10]) with the MSRT group attending 89.29 % of sessions and the ERT group attending 85.42 % of sessions. This is particularly promising as participants in the Horan [10] and Robert’s [48] studies received compensation after attending each training session whereas we did not provide such compensation and reimbursed only a small amount of money ($15AUD) for each assessment session; thus, our results might more closely approximate what could be expected in real world clinical settings. We specifically designed the games and activities that comprise the SoCog programs to afford high levels of intrinsic enjoyment and we attribute the high attendance rates to this feature of the training. Scores on the IMI-SR [31] indicated that participants in both types of training enjoyed the program, felt they had a choice about participating, and perceived some value in treatment.

### Limited efficacy

The SoCog-ERT group showed delayed improvements at 3-month follow-up in abilities to recognise dynamically presented negative facial expressions of emotion (i.e., 2 exemplars each of sad, angry, fearful, and disgusted). There was a trend toward improvements immediately after training. Although only 6 participants remained in SoCog-ERT at T3, 5 of these 6 individuals showed moderate increases in accuracy (16.25 % mean increase in accuracy; effect size adjusted for small sample = .539) 3 months after training. This is consistent with the results of our previous brief emotion recognition training studies where we also found delayed improvements 1-month post-training and speculated that was due to participants using newly learned skills in their everyday life such that practice over time led to improvements that were not immediately apparent [7]. CIs showed a wide range of improvements from .25 (3.12 %) to 2.42 (30.21 %). Promisingly however this result is consistent with the magnitude of improvements found in other social cognitive remediation studies (e.g., [10, 11, 48]).

As predicted, the SoCog-MSRT group did not show improvements in emotion recognition accuracy for basic emotions and indeed this group showed a decrease in accuracy at 3-month follow-up. As this type of training did not provide explicit training about how to recognise basic facial expressions this finding might indicate that it is important to provide specific ERT in order to improve these more basic perceptual aspects of social cognition. However, further investigation in a larger study would be needed to draw any definitive conclusions.

Both the ERT and MSRT groups showed an increased ability to recognise complex emotions from static photos of the eyes (RMET) at 3-month follow-up. With the exception of 2 participants in MSRT, all participants showed delayed improvement at 3 months. CIs showed comparable changes for both groups between 1.87 and 5.2 for MSRT and from 1.12 to 5.2 for ERT with large and moderate effect sizes, respectively.

Both training groups showed immediate post-training improvements on false-belief reasoning (PSTFB). These improvements were not durable for the SoCog-MSRT group at 3-months but were for SoCog-ERT. Nevertheless, inspection of the paired data and the width of the CIs for this ERT group contrast suggest the result might be imprecise as most participants showed decreased scores, and only 1 showed no change. This imprecision is most likely due to the small sample size but might also have been influenced by task difficulty. Participants were sometimes reluctant, or refused, to do this task a second and third time because they found it too challenging, therefore follow-up results might also be confounded by tolerability and/or missing data [46].

The SoCog-MSRT group showed reduced biases in a small subset of participants who showed a personalising bias (defined as = > .5, [37]) at baseline. The effect size for this result, adjusted to account for the small sample size, was strong (d_unb_ = 2.26) however the CI was wide indicating the need for replication in a larger sample before strong conclusions can be drawn. Nevertheless, this outcome is encouraging, because the evidence to date that attributional biases can be successfully remediated is variable with few studies showing success in remediating these complex cognitive processes [4, 47].

### Clinical significance and limitations

An important issue that we cannot address directly in this study is whether the results have clinical significance. Whilst the limited efficacy found in this study is encouraging, we experienced some significant challenges in conducting the study that prevent us from drawing any firm conclusions about clinical significance. These challenges related to working with some of the treating staff who were reluctant to have their patients randomised to a control group, the rejection of the initial active control treatment by the participants randomised to that arm of the study and drop-out. All of these contributed to the small sample size that restricted analyses and also precludes us from ruling out practice effects on some of our dependent variables. Our assessing staff were not blind to the allocation of the subjects, which may also have led to an overestimation of treatment effects [49]. The study recruited principally from an inpatient group of participants, who once discharged, were very reluctant to return to the hospital for follow-up testing. Greater mobility of staff and a shorter more concise testing battery would help with participant retention. Further dropouts resulted from group cancellations because outpatient clients in a community service for young people with a psychotic illness were not attending group despite efforts by collaborating clinical staff to encourage attendance. Similar motivational challenges were encountered in another Australian study that used the Social Cognition and Interaction Training program [50] leading those authors to conclude that there is an urgent need to identify the characteristics that distinguish between individuals with schizophrenia who engage with psychosocial programs and those who do not (e.g., active positive symptoms and level of neurocognitive impairment). These factors are important both for keeping participants in treatment and for ascertaining who might benefit from treatment and who will not. Our experience, together with Parker et al.’s, highlights the necessity of large multisite studies to increase power and to investigate treatment moderators and mediators.

Our results also highlight the difficulties inherent in conducting randomised controlled trials within real-world clinical settings. We found that both consumers and clinicians were often not responsive to randomisation into a control group. This is because most services encourage active engagement in a range of rehabilitation programs. Thus, clinicians and consumers alike can see 3−12 months (or more) of participation in the control group of a longitudinal RCT as a potential impediment to the rehabilitation process.

RCTs are considered the gold standard test for new treatments [51]; however, in the case of psychosocial remediation, adherence to this RCT model might well impede our ability to establish strong evidence-based treatments. Williams [51] provides a thought provoking discussion of the perils of RCTs, noting that the results can only inform about group outcomes, but do not help distinguish which participants will engage with, and benefit from, a treatment, from those who will not. In addition, RCT outcomes based on null hypothesis testing assume that participants are similar in how they respond to treatment, when in reality treatment will vary as a result of individual differences (e.g., baseline cognitive abilities and/or negative symptoms and/or baseline levels of social interaction). As such, only a subset of participants randomised into a treatment group would be expected to benefit from (or indeed engage with) treatment. This heterogeneity can significantly reduce the power of a study to find treatment effects, and has a profound impact on the generalisability of results [51]. The pragmatic difficulties experienced in this study, and by Parker et al. [50] in their study, highlight the perils of RCTs discussed by Williams. Thus, a more achievable alternative for testing psychosocial treatments in schizophrenia might be a personalised treatment protocol wherein participants are allocated into treatment based on predetermined individual characteristics, with any within-subject differences over time used to index treatment response (rather than comparison with a ‘control group’). For example, we have found that working memory seems important in predicting outcomes from ERT [7, 8] therefore participants with severe working memory deficits might not be expected to benefit from social cognitive treatment before undertaking neurocognitive training. This would potentially increase the power of such studies by as much as 70 % [51, 52].

## Conclusions

Despite the above limitations, the results of this acceptability and feasibility study are encouraging and are consistent with studies testing similar programs. Importantly, participants reported being motivated to participate, enjoying the program and perceiving it as valuable thus supporting the acceptability and feasibility of SoCog. Limited efficacy was observed despite the significant chronicity of many of the participants. Perhaps most promising was that we were able to work closely over several years with hospital staff to implement SoCog within an existing inpatient rehabilitation program. As such, the difficulties that we encountered arguably resemble more closely clinical practice, thus, our results are likely similar to what a clinical team might expect using SoCog. Moreover, SoCog is now run as part of standard care (i.e., outside of a research study) in several mental health services across Australia by clinicians who continue to provide feedback for further development and refinement of the program. The results also suggest that basic emotion recognition might not improve without specific training.

## Footnotes

1 The original testing session was taking in excess of 3 h and participants were becoming very fatigued therefore, we needed to cut some tests to avoid fatigue effects on outcomes. The IPSAQ was chosen due to its length; therefore, we have limited data on this measure.
